# Infrared thermal imaging reveals distinct patterns in adolescent anterior knee pain: A pilot study

**DOI:** 10.1002/jeo2.70478

**Published:** 2025-11-14

**Authors:** Ali Yalcinkaya, Michael Skovdal Rathleff, Jakob Khezr, Jens Svendsson, Hans‐Christen Husum, Ole Rahbek, Søren Kold, Shima Gholinezhad

**Affiliations:** ^1^ Department of Orthopaedics Aalborg University Hospital Aalborg Denmark; ^2^ Department of Health Science and Technology Aalborg University Aalborg Denmark; ^3^ Department of Radiology Randers Regional Hospital Randers Denmark; ^4^ Department of Orthopaedics Aarhus University Hospital Aarhus Denmark; ^5^ Department of Clinical Medicine Aalborg University Aalborg Denmark

**Keywords:** anterior knee pain, infrared thermography, Osgood–Schlatter disease, patellofemoral pain, pediatric orthopaedics

## Abstract

**Purpose:**

The two most common types of anterior knee pain (AKP) in adolescents are patellofemoral pain (PFP) and Osgood–Schlatter disease (OSD). Accurate diagnosis is essential to guide treatment, but can be challenging due to overlapping clinical features of these two conditions. Infrared thermography (IRT) offers a noninvasive method to detect physiological differences such as local inflammation or altered perfusion. The aim of this exploratory study was to evaluate whether IRT can detect distinct thermal profiles in adolescents with unilateral PFP or OSD and explore its potential as a supplementary diagnostic tool.

**Methods:**

This within‐subject study included 28 adolescents with unilateral AKP (17 with PFP, 11 with OSD) who underwent infrared thermography. Mean skin temperatures were recorded for both symptomatic and contralateral knees, and side‐to‐side differences were calculated for each participant. These individual asymmetries were then summarised descriptively within each diagnostic group and compared between groups.

**Results:**

In the OSD group, symptomatic knees exhibited higher skin temperatures than contralateral knees (mean ΔT = +0.48 ± 0.47°C 95% confidence interval [CI]: 0.16–0.80), whereas in the PFP group, symptomatic knees showed lower temperatures (mean ΔT = −0.78 ± 90°C, 95% CI: −1.25 to −0.32). The between‐group contrast corresponded to a large standardised mean difference (Hedges' *g* = 1.58, 95% CI 0.71–2.45), indicating distinct condition‐specific thermal patterns.

**Conclusion:**

Infrared thermography revealed contrasting thermal asymmetries in adolescents with PFP or OSD. These preliminary findings suggest that IRT may help characterise distinct thermal patterns associated with AKP.

**Level of Evidence:**

Level III.

**Abbreviations**
AKP: anterior knee pain
IRT: infrared thermography
OSD: Osgood–Schlatter disease
PFP: patellofemoral pain
RoI: region of interest
ΔT: within‐subject asymmetry

## INTRODUCTION

Knee pain affects approximately one in three adolescents, making it one of the most common sites of musculoskeletal pain in this age group [8]. The most frequently diagnosed anterior knee pain (AKP) conditions are patellofemoral pain syndrome (PFP) and Osgood–Schlatter disease (OSD) [20].

Emerging evidence indicates that OSD occurs during periods of rapid growth and repetitive mechanical loading. This inflammatory response is associated with increased vascular activity in the affected region [24]. In contrast, PFP is characterised by more complex and heterogeneous mechanisms, with patients exhibiting variable physiological responses [9]. Among these, some studies have identified signs of hypoperfusion and vascular dysregulation, suggesting that vascular factors may underlie symptoms in at least a subset of individuals with PFP [7, 16].

A specific diagnosis is essential to guide targeted treatment strategies and to provide validation and reassurance to adolescents [10]. OSD typically presents as localised pain, swelling and tenderness at the tibial tuberosity [3], while PFP is associated with diffuse AKP, often described as aching discomfort around or behind the patella [19]. While patient‐reported outcome measures offer insight into the patient's subjective experience, they rely on self‐report and can introduce variability [17].

Given a demand for more objective and noninvasive examination methods, infrared thermal imaging (IRT) has emerged as a promising adjunct in musculoskeletal assessment [12]. IRT detects infrared radiation emitted from the skin to measure surface temperature [11], offering a noncontact method for capturing physiological changes in tissue. Recently, IRT has been increasingly explored in the assessment of knee disorders, with a growing body of literature, particularly in conditions such as knee osteoarthritis [4, 14], demonstrating its potential to detect abnormal thermal patterns associated with inflammation and joint degeneration.

This pilot study aimed to evaluate the sensitivity of IRT in detecting temperature asymmetries over the anterior knee in adolescents with AKP. In this study, it was hypothesised that OSD would be associated with increased local skin temperature due to inflammation, whereas PFP would show more variable patterns, potentially including reduced temperature as a result of hypoperfusion or vascular dysregulation. By comparing symptomatic and contralateral knees within individuals, the study examined whether IRT could distinguish between diagnostic subgroups through their thermal patterns.

## METHODS

### Study design and setting

This was a prospective cross‐sectional study conducted at Aalborg University Hospital. The protocol was registered with the Region Nordjylland, Danish Data Protection Agency (File: 2021‐025). The local ethical committee determined that the project did not fall within the definition of a notifiable health research project, as specified by the Committee Act (Consolidated Act no. 1083 of 15/09/2017).

### Participants

The study included adolescent patients referred to the Orthopaedic Surgery Department with AKP from 2020 to 2021. Inclusion criteria were a diagnosis of either OSD or PFP with unilateral knee pain and willingness to participate in the study. No exclusion criteria were applied. All participants were evaluated at their first clinical visit, and no treatment had been initiated at the time of assessment. None of the participants had a history of previous knee surgery or recent trauma.

### Clinical evaluation

Diagnoses were confirmed through clinical examination and patient history, using diagnostic criteria consistent with those described by Rathieff et al. and Sorensen et al. [20, 25]. In this cohort, OSD was diagnosed by localised pain and tenderness at the tibial tuberosity, often with swelling, aggravated by activities such as jumping or kneeling during growth spurts. PFP was identified by anterior knee or peripatellar pain, typically persisting for several weeks and provoked by activities such as prolonged sitting, running, or stair climbing. Other potential causes of AKP, such as patellar tendinopathy or meniscal injury, were excluded based on clinical examination.

### Pain mapping

**Figure 1:**
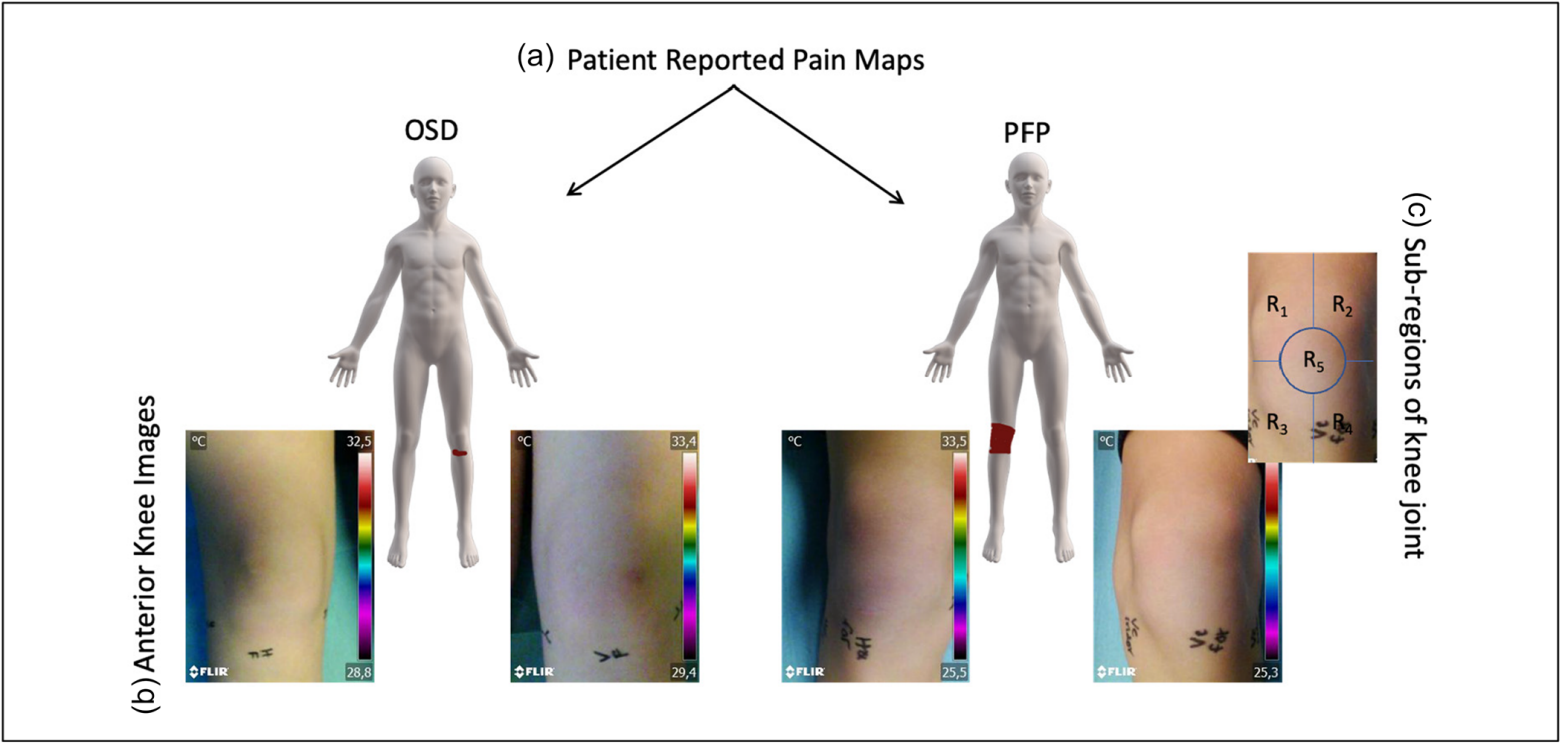
Pain mapping. (a) Representative patient‐reported pain maps from two adolescents, one diagnosed with Osgood–Schlatter disease (OSD) (left) and one with patellofemoral pain (PFP) (right), illustrating typical pain localisation. (b) Corresponding anterior knee photographs of the same patients. (c) Schematic representation of the patella divided into five subregions (R1–R5), used to approximate pain‐specific areas based on patient input.

Participants were asked to illustrate their knee pain areas on a validated, high‐resolution digital body chart, using the Navigate Pain Android app (Version 1, Aalborg University). Drawings were completed on a Samsung Galaxy Note tablet (Android 4.1.2), using a 1.5 mm‐tipped ‘S Pen’ stylus. Following established guidelines, participants were instructed to mark all current pain areas on and around the knee, to shade the painful regions. Figure 1a displays representative patient‐reported pain maps drawn by two patients diagnosed with OSD (left) and PFP (right). Pain distribution patterns were categorised into three main types: Ovate (covering the entire patella region), Hook (curving around the medial or lateral patella) and Anchor (concentrated below the patella towards the patellar tendon insertion), based on the framework proposed by Boudreau et al. [1]. Although this classification was originally developed for PFP, it was applied to both diagnostic groups in the present study to ensure consistent comparison of AKP patterns.

### Thermal imaging

Thermographic imaging was performed using an infrared thermal camera (FLIR T540, FLIR Systems AB, Täby, Sweden) with a measurement accuracy of ±2% of the reading and a thermal sensitivity of <0.03°C. Skin emissivity was set to 0.98, as recommended for human skin.

Prior to imaging, patients exposed their legs and feet and were positioned on an examination table for 10 min to allow skin temperature to acclimatise to ambient room conditions [4, 13]. Ambient room temperature was maintained between 21°C and 23°C. To minimise variation across participants, the camera was positioned approximately 1 m away, capturing the knee from the frontal side, extending from the suprapatellar region to the inferior border of the knee joint (Figure 1b). The camera simultaneously captured a thermal image and a corresponding digital (visible‐light) image. The resolution of the thermal image was 320 × 240 pixels.

For preprocessing, temperature data were extracted using FLIR Research IR software and saved in comma‐separated values (CSV) format. To eliminate background interference, digital and thermal images of the knee were displayed side by side, and the kneecap was manually isolated in the thermal image using anatomical landmarks from the digital image, specifically the patella borders relative to the tibial tuberosity and surrounding soft‐tissue contours. Thermal features were then extracted from two sources: (1) the entire patellar surface and (2) the pain area identified by the participant, approximated by overlaying each drawing onto a standardised anatomical segmentation of the patella into five regions (R1–R5; see Figure 1c). For consistency, the central ROI (R5) was defined as a circle with a radius equal to one‐fourth of the kneecap image width, centred over the patella, with surrounding quadrants (R1–R4) delineated accordingly. This approach allowed each participant′s reported pain to be mapped onto a structured ROI framework. ROI masks were then applied to the thermal images and overlaid with the corresponding temperature data extracted in CSV format. Next, for each participant, skin temperature distributions and mean temperatures were calculated separately for the symptomatic (painful) and contralateral (nonpainful) knees. In this study, it was assumed that, in the absence of pathology, knee temperatures would be symmetrical within each participant [21], and any observed temperature differences between symptomatic and contralateral knees were attributed to pathological conditions.

### Statistical analysis

Descriptive statistics were computed for mean skin temperature values of both symptomatic and contralateral knees, including the mean and standard deviation for the overall patellar region as well as patient‐reported pain‐specific regions. Temperature distributions were visualised using kernel density plots stratified by diagnosis (PFP and OSD) and knee (symptomatic vs. contralateral). Within‐subject temperature differences between symptomatic and contralateral knees (*ΔT; within‐subject asymmetry*) were calculated to quantify thermal asymmetry, and the mean ΔT with 95% confidence intervals (CIs) was reported for the PFP and OSD subgroups. Paired differences were further visualised using boxplots. Group‐level contrasts were summarised using mean differences with 95% confidence intervals and standardised effect sizes (Hedges′ g). All analyses and visualisations were performed in R (version 4.2.2).

## RESULTS

A total of 28 adolescents were included in the study, comprising 17 females and 11 males, with a mean age of 12.7 ± 1.2 years (range: 10–15) and a mean BMI of 19.4 ± 3.0 kg/m² (range: 15.4–25.8). Of these, 11 were diagnosed with OSD and 17 with PFP. The symptomatic knee was on the left side in 19 participants and on the right side in nine participants.

Based on patient‐reported pain mapping, distinct distribution patterns were observed across diagnostic groups. Among the 28 cases, the *Ovate* pattern was most common in PFP (9/17 cases). In contrast, the *Anchor* pattern predominated in OSD (10/11 cases), with one additional Anchor case reported in a participant with PFP. The *Hook* pattern was observed more frequently in PFP (seven cases) compared with OSD (one case).

Temperature measurements were obtained from two sources: the entire patellar region and the pain‐specific areas identified by each participant. These values were highly correlated (*r*² = 0.91). Therefore, to better reflect each individual's reported pain location, all further analyses were based on temperatures extracted from the pain‐mapped regions.

When averaged across all participants (pooling both groups), the mean temperature of the symptomatic knee was 30.69 ± 1.39°C, compared with 30.98 ± 1.16°C in the contralateral knee. The resulting Δ*T* was −0.29°C (95% CI: −0.67 to 0.09), indicating a slight trend towards lower temperatures in symptomatic knees.

**Figure 2:**
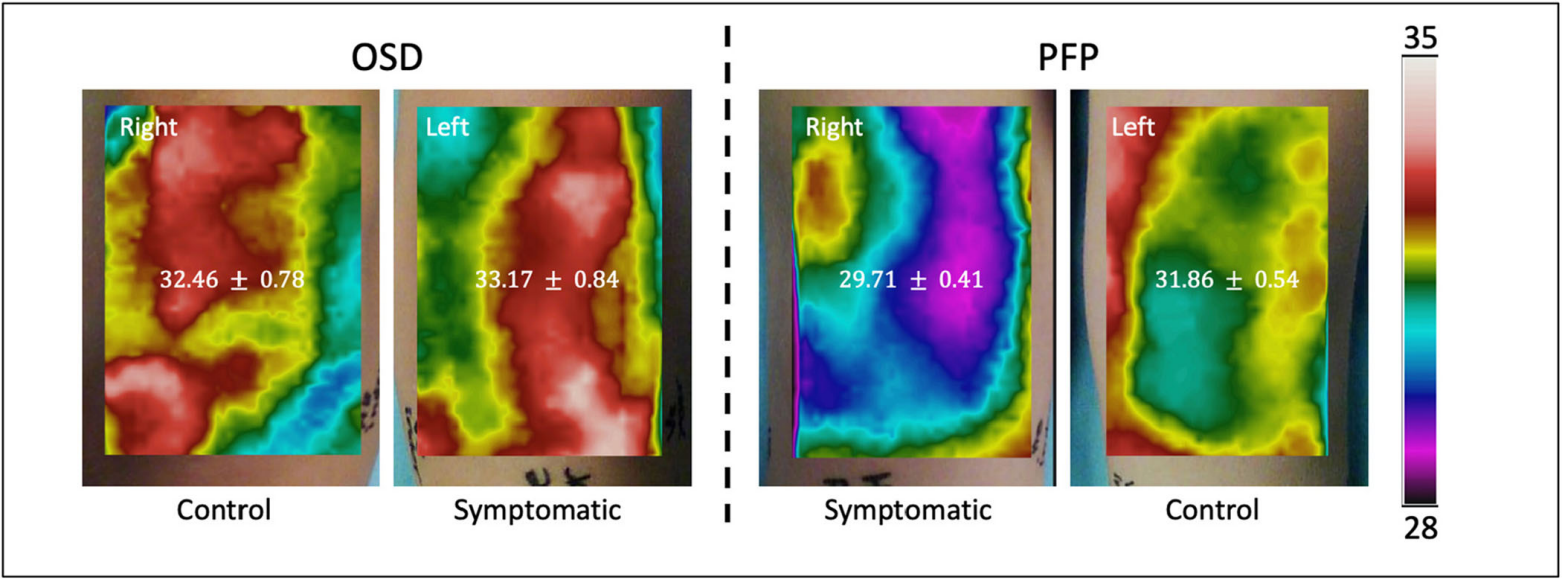
Representative thermal images from adolescents with Osgood–Schlatter disease (OSD) (left) and patellofemoral pain (PFP) (right), illustrating temperature distributions in symptomatic and contralateral knees.

Figure 2 presents representative thermal images from two participants with different diagnoses: the left image shows a case of OSD affecting the left knee, and the right image shows a case of PFP affecting the right knee. The participant with OSD exhibited higher mean knee temperatures than the participant with PFP. Specifically, in the OSD case, the mean temperature of the symptomatic (left) knee was 33.17°C, compared with 32.46°C in the contralateral (right) knee. In contrast, in the PFP case, the symptomatic (right) knee had a lower mean temperature (29.71°C) than the contralateral (left) knee (31.88°C).

**Figure 3:**
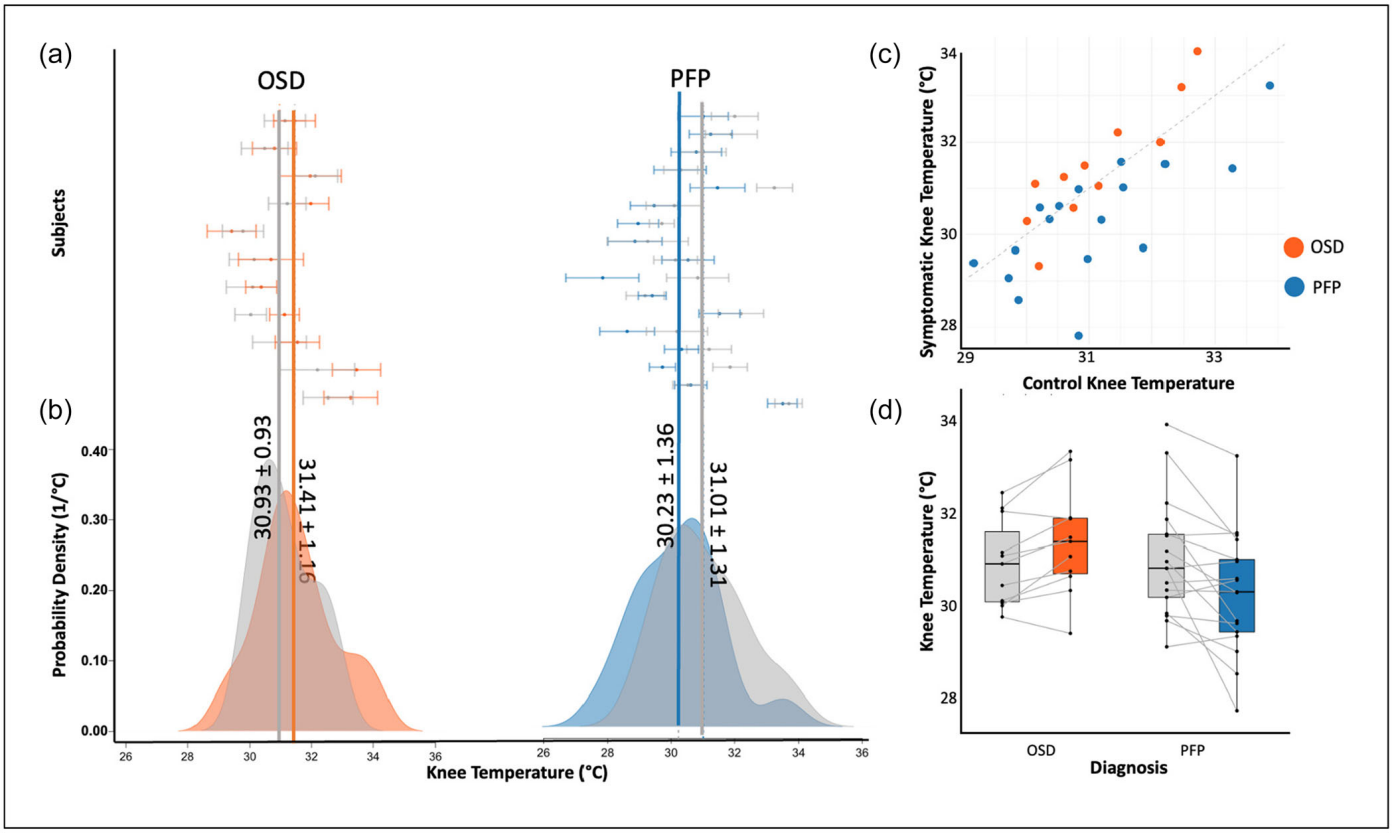
Thermographic comparisons of symptomatic and contralateral knees in Osgood–Schlatter disease (OSD) and patellofemoral pain (PFP). (a) Individual‐level plots of mean skin temperature in symptomatic knees (OSD: orange, PFP: blue) and contralateral knees (grey). (b) Kernel density distributions of symptomatic and contralateral knee temperatures in OSD (left) and PFP (right). (c) Scatter plot of symptomatic versus contralateral knee temperatures, coloured by diagnosis, with the dashed diagonal indicating equality. (d) Paired boxplots of mean skin temperature in symptomatic and contralateral knees for OSD and PFP.

This pattern was consistent across participants, as illustrated in Figure 3, which presents paired comparisons of symptomatic and contralateral knees in each group. Figure 3a displays individual knee temperatures with mean values and standard deviations for symptomatic (coloured) and contralateral (grey) knees. Figure 3b shows the corresponding group‐level density distributions, highlighting the shift towards higher symptomatic knee temperatures in OSD and lower symptomatic knee temperatures in PFP. In the OSD group, symptomatic knees averaged 31.41°C ± 1.16°C compared with 30.93°C ± 0.93°C in contralateral knees. In contrast, the PFP group exhibited lower symptomatic knee temperatures (30.23 ± 1.36°C) compared with contralateral knees (31.01°C ± 1.32°C). The scatter plot in Figure 3c highlights this separation: most OSD participants cluster above the line of equality, whereas most PFP participants cluster below. Finally, the paired boxplots in Figure 3d illustrate that 9 of 11 OSD participants had higher symptomatic knee temperatures, while 13 of 17 PFP participants had lower symptomatic knee temperatures relative to their contralateral side.

**Figure 4:**
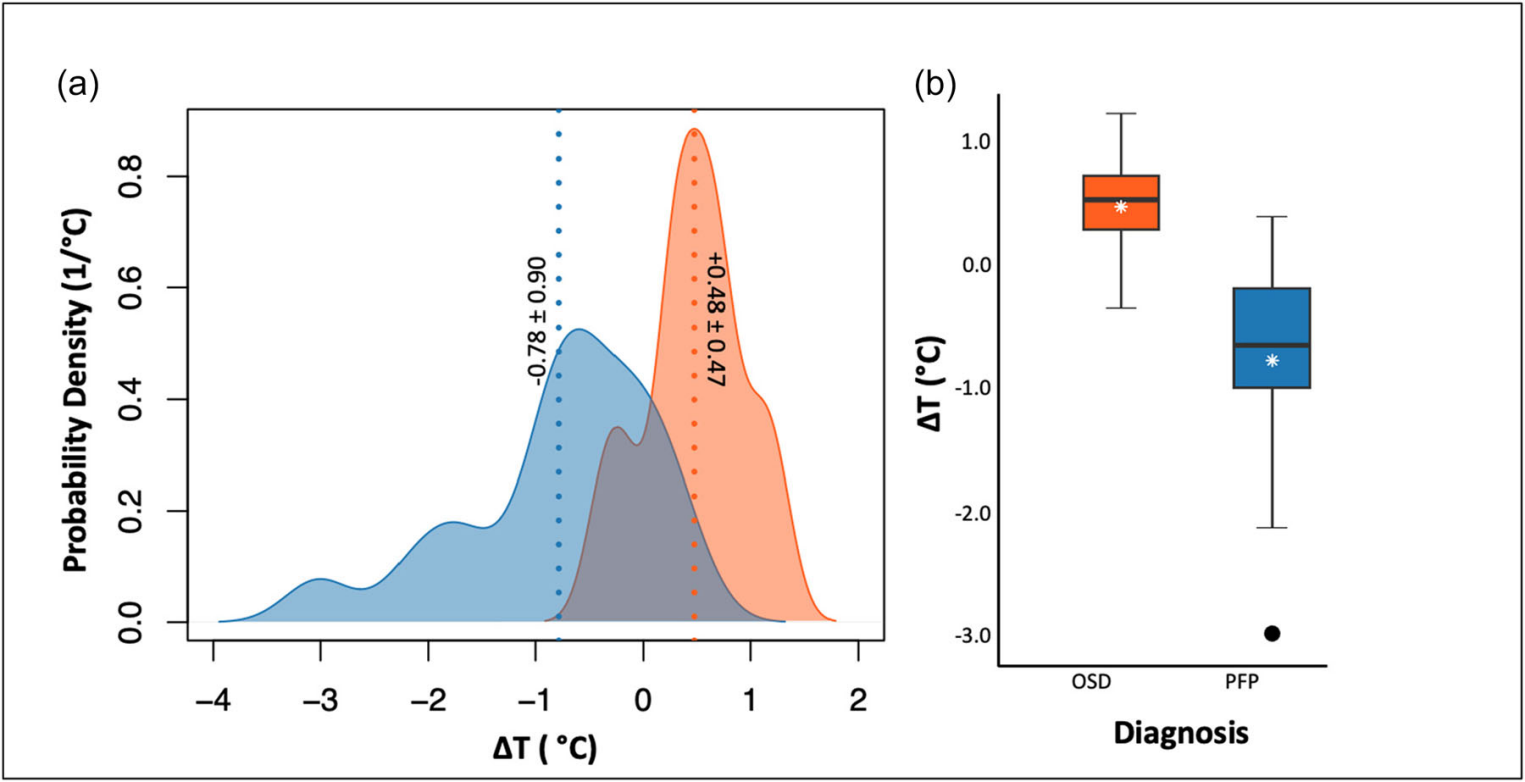
Thermographic comparison of thermal asymmetry (Δ*T*). (a) Kernel density estimates of Δ*T* for Osgood–Schlatter disease (OSD) (orange) and patellofemoral pain (PFP) (blue), with vertical dotted lines marking group means (mean ± SD shown). (b) Boxplots of ΔT distributions by diagnosis (OSD, *n* = 11; PFP, *n* = 17). White stars indicate group means, and black circles denote individual outliers.

Figure 4 illustrates between‐group differences in thermal asymmetry (Δ*T*). The kernel density plots (panel a) highlight opposing distributions: OSD participants showed positive Δ*T* values, indicating warmer symptomatic knees, whereas PFP participants showed negative Δ*T* values, indicating cooler symptomatic knees. The boxplots (panel b) further summarise these findings, with mean Δ*T* values of +0.48 ± 0.47°C (95% CI: 0.16–0.80) for OSD and −0.78°C ± 0.90°C (95% CI: −1.25 to −0.32) for PFP (Hedges' *g* = 1.58, 95% CI: 0.71°C2.45).

## DISCUSSION

This study extends the current body of knowledge by directly comparing two common causes of AKP in adolescents, OSD and PFP, through the assessment of anterior knee temperature using IRT in adolescents with unilateral symptoms. The key finding was the presence of opposing thermal asymmetries: symptomatic knees in OSD patients exhibited higher skin temperatures, while those with PFP demonstrated lower temperatures relative to the contralateral knee. The resulting between‐group difference in thermal asymmetry was approximately 1.26°C (95% CI: 0.63–1.90), suggesting that thermal asymmetry may provide clinically useful information in differentiating these conditions [22].

OSD is a traction apophysitis of the tibial tuberosity that primarily affects adolescents during periods of rapid growth [3]. The tibial tubercle, which serves as the insertion point for the patellar tendon, remains cartilaginous and biomechanically vulnerable throughout this developmental phase. Repetitive loading from high‐impact activities imposes traction stress on the apophysis, resulting in microtrauma, microvascular disruption and localised inflammation [24]. The ensuing inflammatory response and increased vascular activity in the affected region may elevate local skin temperature, thereby rendering thermal imaging a potentially useful noninvasive diagnostic tool. This physiological rationale is also consistent with previous research. For example, Capitani et al. observed significantly elevated skin temperatures in the affected knees of adolescents with OSD (*p* = 0.027; [2]), and Freitas et al. reported similar results, with OSD patients demonstrating higher temperatures compared to age‐matched controls (*p* = 0.008; [23]). Collectively, these findings, together with the present study, support our hypothesis that adolescents with OSD exhibit elevated local skin temperature, indicating the potential of IRT to detect inflammation associated with this condition.

Unlike OSD, PFP involves complex interactions among biomechanical factors, and neuromuscular control. A number of studies suggest that abnormal patellofemoral joint loading and repetitive mechanical stress may lead to microvascular disturbances, and regional hypoperfusion, particularly in the patellar subchondral bone [7, 16, 26]. For instance, Hansen et al. used sodium fluoride PET imaging to investigate bone turnover and perfusion in patients with unilateral PFP [7]. Significantly reduced perfusion and tracer uptake were observed in the superficial patella of symptomatic knees at rest, findings that support the hypothesis of impaired vascular response in PFP. In another study, photoplethysmography has been employed to assess pulsatile blood flow in the patella in a clinically feasible manner. Following passive knee flexion from 20° to 90°, patients with PFP exhibited a significant decrease in patellar blood flow, whereas healthy controls demonstrated no consistent change, further supporting the concept of vascular dysregulation in PFP [16]. These studies, along with our own findings, point towards a possible of altered vascular responses, particularly hypoperfusion, though emerging evidence suggests this pattern may not be uniform across all individuals.

However, the heterogeneity among patients with patellofemoral pain is unavoidable. Trejo‐Chávez et al.   [26] reported no significant baseline temperature differences between healthy individuals and patients with bilateral PFP. In contrast, the present study demonstrated a decreasing trend in symptomatic knee temperature relative to the contralateral side in 13 of 17 PFP patients. This apparent discrepancy may be explained, at least in part, by more recent evidence indicating that patellar skin temperature in PFP is not uniform but can follow three distinct patterns; cold, normal and hot, within the same diagnostic group [9]. Consistent with this, greater temperature variance was observed in the PFP group (*σ*² = 1.87) compared with contralateral knees (*σ*² = 1.36), supporting the presence of more diverse or unstable thermoregulatory patterns in this population. Although this heterogeneity may reduce the precision of thermal imaging in the current study, it may also point to distinct subtypes or patterns within the PFP population, offering a promising direction for future research.

In the current study, patient‐reported pain maps revealed clear differences in the location and distribution of pain between adolescents diagnosed with OSD and PFP. This distinction is expected, as pain location remains a key component in the differential diagnosis of AKP [20]. Moreover, the pain location in PFP was more variable compared with the consistent presentation observed in OSD. These findings are further supported by Boudreau et al., who identified three recurring pain distribution patterns, among individuals with PFP using digital pain mapping [1]. Despite these spatial differences, no significant temperature differences were detected between patient‐defined pain regions and the broader patellar area. This observation is consistent with previous experimental work demonstrating that sympathetic vasomotor responses, measured via thermography, can produce temperature changes extending beyond the site of reported or experimentally induced pain [5].

This study has several limitations that should be acknowledged. First, the sample size was relatively small, which restricts generalisability; larger cohorts will be required to confirm the observed thermal patterns and to enable more robust subgroup analyses. In particular, given the heterogeneity of PFP, larger samples will be necessary to clarify potential subtypes and underlying mechanisms. Second, in this study, diagnoses were based solely on clinical history and examination, without confirmation by imaging (e.g., MRI or ultrasound), which introduces a potential risk of misclassification. The clinical role of imaging in AKP, particularly in PFP, remains debated: while some authors suggest it may provide insight into underlying mechanisms or subtypes, others emphasise that imaging is rarely necessary for establishing the diagnosis [6]. Third, in the current study, a 10‐min acclimatisation period was used; however, some sources recommend up to 15 min where feasible [15], and future studies may consider adopting this longer interval. Finally, reliability indices such as test–retest agreement and the standard error of measurement could not be determined in this study, as only a single thermographic image was acquired per knee. Nevertheless, previous studies using infrared cameras with substantially lower resolution and sensitivity than the FLIR T540 have reported good to excellent reliability in comparable clinical contexts [18]. While this supports the expectation of similar or better reliability in the present protocol, this assumption requires confirmation in future work. Taken together, these limitations should be considered when interpreting the findings.

## CONCLUSION

In conclusion, this exploratory pilot study identified contrasting thermal asymmetry patterns in adolescents with OSD and patellofemoral pain, and provides promising preliminary evidence that infrared thermography can reveal distinct condition‐specific thermal patterns associated with AKP. These findings may suggest potential diagnostic relevance, but confirmation in larger cohorts is required before any clinical implications can be drawn.

## AUTHOR CONTRIBUTIONS

**Ali Yalcinkaya**: Data acquisition; methodology development and implementation; draft manuscript preparation. **Michael Skovdal Rathleff**: Study conception and design; interpretation of results. **Jakob Khezr**: Methodology development and implementation. **Jens Svendsson**: Data acquisition. **Hans‐Christen Husum**: Data acquisition. **Ole Rahbek**: Study conception and design. **Søren Kold**: Study conception and design. **Shima Gholinezhad**: Study conception and design; methodology development and implementation; analysis; interpretation of results; draft manuscript preparation. All authors reviewed the results and approved the final version of the manuscript.

## CONFLICT OF INTEREST STATEMENT

The authors declare no conflicts of interest.

## ETHICS STATEMENT

The protocol was registered at Region NordJylland, Danish Data Protection Agency (File: 2021‐025). The project was not deemed by the local ethical committee to fall within the definition of a notifiable health research project as defined by the Committee Act (Consolidated Act no. 1083 of 15/09/2017). Informed consent was obtained from all participants' legal guardians.

## Data Availability

The datasets used and/or analysed during the current study are available from the corresponding author on reasonable request.
